# Factors Contributing to Medication Adherence in Patients with a Chronic Condition: A Scoping Review of Qualitative Research

**DOI:** 10.3390/pharmaceutics13071100

**Published:** 2021-07-20

**Authors:** Kirsi Kvarnström, Aleksi Westerholm, Marja Airaksinen, Helena Liira

**Affiliations:** 1 HUS Pharmacy, University of Helsinki and Helsinki University Hospital, 00290 Helsinki, Finland; 2 Clinical Pharmacy Group, Division of Pharmacology and Pharmacotherapy, Faculty of Pharmacy, University of Helsinki, 00014 Helsinki, Finland; aleksi.westerholm@helsinki.fi (A.W.); marjaairaksinen@gmail.com (M.A.); 3 Department of General Practice, University of Helsinki, 00290 Helsinki, Finland; helena.liira@helsinki.fi; 4 Unit of Primary Health Care, University of Helsinki and Helsinki University Hospital, 00290 Helsinki, Finland

**Keywords:** medication adherence, patient compliance, primary non-adherence, patient-related factors, qualitative research, barriers, facilitators, scoping review, chronic conditions

## Abstract

Introduction: Medication adherence continues to be a significant challenge in healthcare, and there is a shortage of effective interventions in this area. This scoping review studied the patient-related factors of medication adherence. Methods: We searched Medline Ovid, Scopus, and Cochrane Library from January 2009 to June 2021 to find the most recent original qualitative studies or systematic reviews that addressed the patient-related factors of medication adherence in treating chronic conditions. We used the PRISMA-ScR checklist to ensure the quality of the study. Results: The initial search revealed 4404 studies, of which we included 89 qualitative studies in the scoping review. We inductively organized the patient-related factors causing barriers, as well as the facilitators to medication adherence. The studies more often dealt with barriers than facilitators. We classified the factors as patient-specific, illness-specific, medication-related, healthcare and system-related, sociocultural, as well as logistical and financial factors. Information and knowledge of diseases and their treatment, communication, trust in patient-provider relationships, support, and adequate resources appeared to be the critical facilitators in medication adherence from the patient perspective. Discussion and conclusions: Patients are willing to discuss their concerns about medications. Better communication and better information on medicines appear to be among the critical factors for patients. The findings of this scoping review may help those who plan further interventions to improve medication adherence.

## 1. Introduction

Medication adherence continues to be a significant challenge in healthcare, and there is a shortage of effective interventions. In 2003, the World Health Organization identified that only 50% of chronically ill patients take their medication as prescribed in developed countries [1]. Although there is a wealth of controlled trials on interventions to improve adherence, current methods of improving medication adherence for chronic health problems are mostly complex and not effective [2,3,4]. Previous studies and systematic reviews have combined the existing evidence of adherence interventions [3]. Nevertheless, it seems that there is still a lack of understanding about the complexity of medication adherence from the patient’s perspective.

Medication nonadherence is associated with poorer health outcomes [5]. If patients do not gain the expected health benefits from their medication because of nonadherence, the burden of health care costs increases for both patients and society in general [6,7,8]. The same factors that improve medication adherence may also decrease it [9,10,11]. The patient can experience medication-related burdens, which may negatively affect adherence [12]. On the other hand, a patient’s nonadherence can be seen as a behavioural problem related to their course of action [13]. Many studies have focused on medication adherence related to some specific illness instead of medication adherence in general. The studies may be lacking the input of patients, while the viewpoint of healthcare professionals may have been dominant. Patients struggle in reconciling daily life with comorbidity and multiple medications may be poorly understood [14]. Patient-centred care requires a greater understanding of the daily decisions patients need to make in order to manage a complex medication regimen.

Many theories have been applied to explain medication adherence behaviour. The information–motivation–behavioural skills (IMB) model is a widely used social behaviour model to explain medication adherence among chronically ill patients [15,16]. According to the model, the following three dimensions influence adherence behaviour: (1) Information and knowledge about the need for essential behaviour, (2) Motivation to make necessary behavioural changes and (3) The required behavioural skills to achieve the desired behaviour.

The model may explain patients’ actions regarding their rational use of medicines. Patients may not have sufficient information and understanding about their illness or medication to make an adequate decision, and they can seek the information from various sources [17]. Patient motivation is crucial to cope with multiple medications and make these fit into daily life. On the other hand, there may be personal reasons and system and organisation-specific barriers, which can lead to unwanted behaviour and medication nonadherence [18]. However, no theory alone seems to explain a patient’s adherence to medication because there may be external factors that can also affect adherence.

We need a more patient-focused approach to medication adherence and a better understanding of this complex phenomenon. This scoping review aimed at a better understanding of patients’ views on medication adherence and analysing the contributing factors as to why patients are not taking the medication as prescribed in outpatient settings. We wanted to understand this complex phenomenon in depth and summarize our findings in this scoping review.

## 2. Materials and Methods

We used the PRISMA-ScR checklist to ensure the quality of the study. The present scoping review is reported based on the guidelines of Preferred Reporting Items for Systematic reviews and Meta-Analyses extension for Scoping Reviews [19]. The PRISMA-ScR is available from the authors upon request.

### 2.1. Search Strategy

The literature search for eligible qualitative studies was conducted on 23 September 2019, using MEDLINE (Ovid), Scopus, and the Cochrane Library, with the assistance of an information specialist at the Helsinki University Library. The search was updated on 9 June 2021. We included articles that were published from January 2009 to June 2021. We wanted to focus on the most recent publications, so we did not include the publications published before the year 2009. We limited the article search to English language studies and articles published in peer-reviewed journals. We used the following wide range of search terms related to medication, drug, medicine, adherence, non-adherence, compliance, non-compliance, patient, experience, fear, beliefs, knowledge, attitudes, behaviour, communication, reason, and cause. Relating to the study design, our search terms were: qualitative, interview, focus group, questionnaire, observation, study, and research. An example of the search strategy is presented in the included appendix material (Appendix A).

### 2.2. Inclusion and Exclusion Criteria

We were interested in the phenomena leading to medication adherence and non-adherence from the patient perspective. Therefore, we included qualitative studies where the primary focus was understanding the complexity of medication adherence described by patients who were being treated for chronic conditions. We included original qualitative studies and systematic reviews if the study population consisted of people of 18 years or older and patients with at least one chronic condition. We also required that the primary focus was on patients’ experiences and attitudes towards medication adherence. We did not require comparison groups. It was mandatory that the researchers had used qualitative methods both for data collection and data analysis. We wanted to study the phenomena in general, so we excluded studies where the primary study population consisted of children or adolescents under 18 years or patients with an acute illness who were pregnant or drug or alcohol users. We also excluded conference papers, quantitative methods and mixed methods studies, as well as studies that collected data using qualitative methods, but data was analysed using quantitative methods.

### 2.3. Study Selection

The systematic searches for eligible articles retrieved 4404 studies. After duplicates were removed, the researchers (KK, AW, HL) independently screened the titles and abstracts for eligibility using the online software, Covidence. If one or two reviewers identified the article as relevant, we carried out a full-text review. We solved any disagreements via discussions and reaching a consensus. After the title and abstract screening, two reviewers (KK, AW) independently screened the full text of selected articles. Disagreements were resolved through discussions with the third reviewer (HL) for final inclusion. The articles were selected in several parts, which allowed the reviewers to have a regular discussion of the eligibility criteria, ensuring the same understanding of the criteria, and the criteria remaining the same throughout the article selection phase.

We did not assess the risk of bias of the included studies. As in many scoping reviews, the goal was to describe the phenomena surrounding patients and medication adherence [20].

### 2.4. Data Extraction

We constructed a template to carry out the data extraction using the Covidence online platform. Two reviewers (KK, HL) independently extracted the data, and the results were reviewed and verified by both reviewers for quality and clarity. We resolved the discrepancies by discussions and reaching a consensus. The data extraction template first focused on the study design, illness, context and concept of the studies, as well as barriers and facilitators to medication adherence. After extracting a third of the studies, we constructed a more specific classification for barriers and facilitators to medication adherence and re-extracted the material from the beginning with the wider list of items. We elaborated this classification further during the analysis of the results. We noted patients’ knowledge of their illness and its treatment. Motivation and behaviour skills seemed to be essential and correlated to good medication self-management during the analysis. Therefore, we decided to apply the IMB model as part of the classification of facilitators to medication adherence [15,16]. The authors provide by request the final list of data items documented in the Covidence extraction form.

## 3. Results

**Figure 1:**
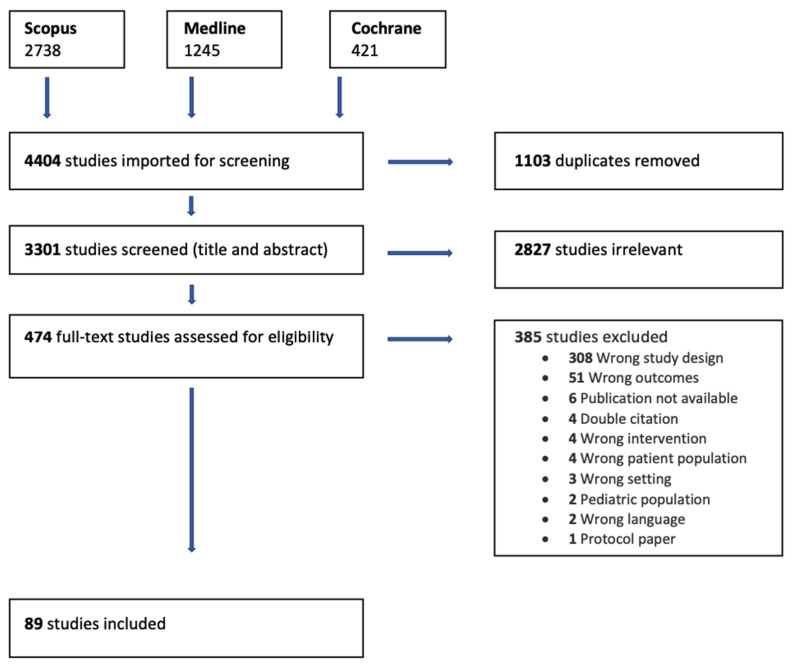
PRISMA flow diagram of the study selection process.

We included 89 original peer-reviewed articles in this scoping review (Figure 1). The study design in all the articles was qualitative and carried out in community or outpatient settings (Table A1). The studies were conducted in 36 different countries: The United States (*n* = 19), The United Kingdom (*n* = 10), South Africa (*n* = 4), Australia (*n* = 3), Canada (*n* = 3), Malaysia (*n* = 3), The Netherlands (*n* = 2), Sweden (*n* = 2), Indonesia (*n* = 2), Iran (*n* = 2) and one study from each of the following countries: Belgium, Norway, Portugal, Spain, Switzerland, Germany, Ireland, France, Italy, Singapore, New Zealand, Taiwan, Jordan, Pakistan, Kuwait, Saudi-Arabia, Vietnam, Uganda, Tanzania, Kenya, Eswatini, Ethiopia, Namibia and Lesotho. There was one study where both Nepal and Australia were involved and one study where Italy, Portugal and Poland were involved.

Our review covered 13 systematic reviews on medication adherence (Table A2). Seven of them focused on patients with cardiovascular disease or type two diabetes [21,22,23,24,25,26,27], one on patients with rheumatoid arthritis [28], one on patients with breast cancer [29], two on patients with chronic kidney disease or kidney transplants [10,30] and two with no specific illness [31,32].

There were 17 studies that had a behaviour theory-based approach to medication adherence (Table 1). The theories that appeared were: Andersen’s Behavioural Model [33,34], Roy Adaptation Model [35], Common-Sense Model of Self-Regulation [36], Social-Ecological Model [37,38], Therapeutic Alliance [39], Dowell’s Therapeutic Alliance Model and Leventhal’s Common Sense Model [40], Health Literacy Pathway Model [41], ABC Taxonomy and Three-Factor Model [32], Health Belief Model [42,43,44,45], Naturalistic Decision Model [46] and Stages of Change Model [47]. One of the studies did not have a theory-based approach in the beginning, but many of the findings fitted together with the Information–Motivation–Behaviour model [48].

The context of most of the studies was an outpatient setting, either in primary or secondary care (Table A1). The studies’ concept varied from the rationale of taking medication to understanding patients’ beliefs, practices, and reasons for nonadherence.

### 3.1. Barriers to Medication Adherence

**Figure 2:**
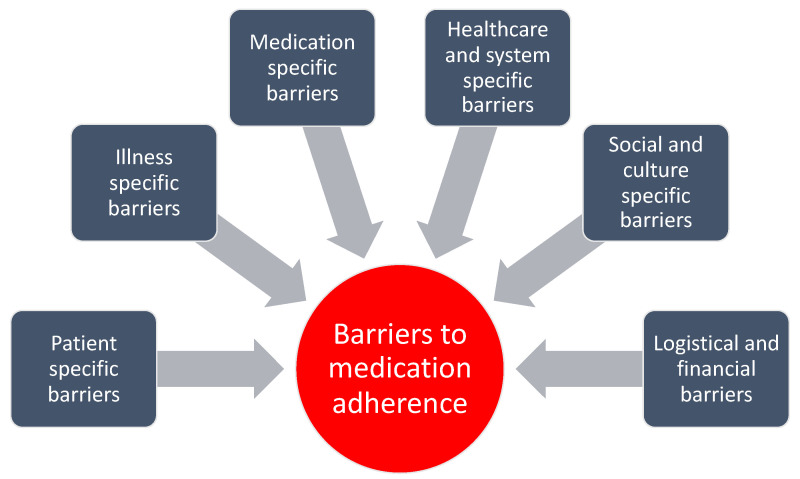
The identified key barriers to medication adherence based on the included qualitative studies (*n* = 89).

**Figure 3:**
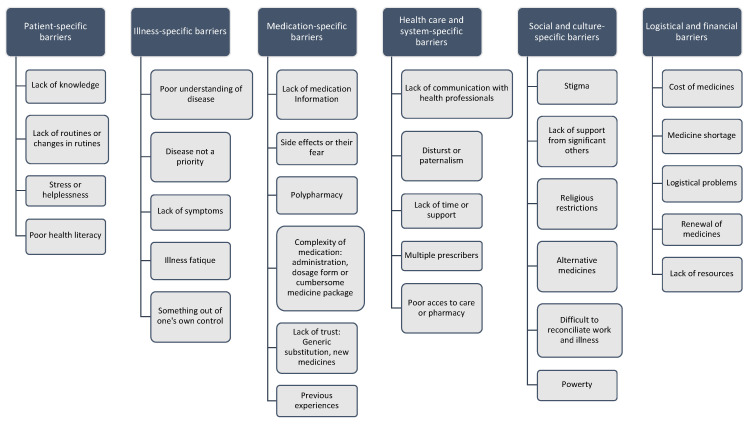
Subcategorisation of barriers to medication adherence arising from the included qualitative studies (*n* = 89).

Overall, the studies reported more barriers than facilitators to medication adherence. We inductively identified six subject areas with subcategories related to barriers to medication adherence based on the included qualitative studies (*n* = 89). The classification was data-driven, and we compiled it after extracting evidence from a third of the studies. We then went back to the beginning and re-extracted the data with the improved categorization. See Figure 2 and Figure 3.

#### 3.1.1. Patient-Specific Barriers

Patients may lack information or knowledge to understand their medication regimen properly. At the beginning of their disease, they may have received medication information and adherence counselling but without any follow-up, leading to patients being forgetful [49]. If the patient is extremely ill at the time of counselling, it may be challenging to adapt the information provided, and misunderstandings can occur. Patients can have poor awareness about the need to take medication as prescribed, and they tend to adjust their doses according to their understanding [46,50]. They may have incorrect or erroneous beliefs about medication [51]. They can lack motivation and think the disease is something they cannot control [52]. A lack of routines, being busy, or changes in practices for special occasions are risk points for medication adherence and can easily lead to missing doses or sleeping through dosing times [48].

Stress and helplessness can affect medication adherence [53]. Injectable drugs may feel unpleasant, and a patient may think injecting will destroy the body [52]. Patients’ physical disabilities can also be a barrier when administrating the medicine, which may require good eyesight or a steady hand [54]. Poor health literacy increases the adherence problem, and there can also be difficulties in understanding written language, especially if it is not written in a patient’s mother tongue [34,41]. Comorbidity may increase the probability of non-adherence [55].

#### 3.1.2. Illness-Specific Barriers

Contrary to healthcare professionals’ expectations, the disease is not always the priority for the patient [35,52,56]. It can be an unwanted episode, but not as important as other matters in life. A patient may have an adverse emotional reaction to the illness and judge life before the illness as more valuable. The required life changes may not be a priority. Patients may also rationalise that the disease is not so severe that they need to take their medication precisely as prescribed. Choosing to take or not to take medicines may depend on how seriously the patient assesses their situation [57].

Sometimes the challenge is that the patient has not accepted the illness or thinks it is someone else’s fault. The negative beliefs of illness or multiple diseases can increase barriers to medication adherence though it can differ from condition to condition [58]. Cancer can be understood as more life-threatening than diabetes, although diabetes can have grave consequences when not treated as required. The disease itself can cause fatigue and overwhelming tiredness, which negatively impact adherence [59].

#### 3.1.3. Medication-Specific Barriers

At the time of the onset of the illness, patients may lack the information on their condition or on the medication they need [60,61]. They can feel confused about the illness duration and prognosis [42,43]. Treatment can often seem time-consuming and complex to them [58,62]. Taking medication can be associated in patients’ minds with being sick, which can negatively influence adherence [35]. Difficulties in integrating medication into daily life can prevent patients from taking medication as prescribed. Working life may require shift work, and night shifts may make it difficult to have regular routines [63]. Besides, the illness may not have visible symptoms, and patients may not feel unwell [64]. Patients also fear that once they start a medication, this means they must continue taking it throughout their life [65].

If the medication information for a patient is inadequate and does not meet patients’ needs, they may use alternative information sources such as the internet [66]. A patient information leaflet in a medicine package may be difficult to understand. Warnings of side effects in the package sometimes make a patient decide not to take the medicine. Generic substitution may cause suspicions of the effect of a generic drug compared with the original product, thereby negatively affecting adherence [37]. Media can also influence opinions of the quality of drugs [67]. The desire of patients to self-regulate their lives may sometimes lead them to use non-prescription drugs instead of prescribed medicines [68].

Struggling with side effects seems to be a common barrier to medication adherence. Fear and the thought of not being safe with their medication may keep patients from taking it [60]. There are also physical barriers surrounding medication-taking: the size of the tablet can make it difficult to swallow, there can be unpleasant metallic after-taste or throat pain [59]. Needle phobia can prevent injecting insulin. A change from oral tablets to injectable drugs can be a drawback for patients [35].

#### 3.1.4. Healthcare and System-Specific Barriers

Poor access to healthcare and long waiting times cause poor medication adherence [55]. Fragmentation of treatment between multiple prescribers, a lack of communication between a general practitioner and a community pharmacist and poor coordination between primary and secondary care can lead to treatment problems. These, in turn, can lead to the discontinuation of care [55,69,70].

A lack of support and empathy from healthcare providers and a paternalistic manner can negatively impact adherence [14,43,55,71]. Poor patient-provider relationships lead to insufficient patient counselling and leave the patient alone struggling with medication problems [43]. Without trust-based patient-provider communication, patients cannot freely discuss side effects and other concerns related to their medication [39,72]. The inability of healthcare professionals to discuss adherence problems with patients and take their concerns and experiences seriously can impact the self-efficacy of patients [73,74]. A lack of trust in doctors and questioning their expertise may increase the burden of the illness and have an essential influence on a patient’s adherence behaviour [60].

#### 3.1.5. Social and Culture-Specific Barriers

A stigma is a common reason for nonadherence, especially with HIV/AIDS and with non-communicable diseases [71]. Patients may not want anybody to know about their illness. The fear of being stigmatized can be so intense that the patient prefers not to take their medication if there is a possibility that someone might be watching. It can be difficult to reconcile work and illness [74]. A lack of support from significant others can have a substantial impact on adherence and control of the illness [75,76].

Patients can prefer traditional alternatives or homeopathic remedies or methods instead of conventional medicine because these are more “natural” [45,72,77]. Patients can have a strong religious faith and prioritize religious rituals instead of taking medicines. Fasting during Ramadan and holy water can have a significant impact on medication management and may be the leading cause to adjust the medication to fit better with religious situations and routines [71]. Patients may stop the medication if they believe that praying can cure them [78].

#### 3.1.6. Logistical and Financial Barriers

Financial burdens and costs of medicines are significant barriers to medication adherence [79]. Unemployment and economic difficulties can affect the ability to buy medicines. If a patient does not have enough money to buy necessities such as food and clothing, medicines are unlikely to be a priority [80]. Difficulties travelling to the clinic, especially in developing countries, can hinder good medication self-management [42]. If insurance coverage is not comprehensive enough or there is no insurance, the cost of medicine can be unbearable [80]. A medicine shortage and the availability of medicines at the clinic or pharmacy, especially in developing countries, can become a significant problem for the continuity of care [60].

### 3.2. Facilitators to Medication Adherence

**Figure 4:**
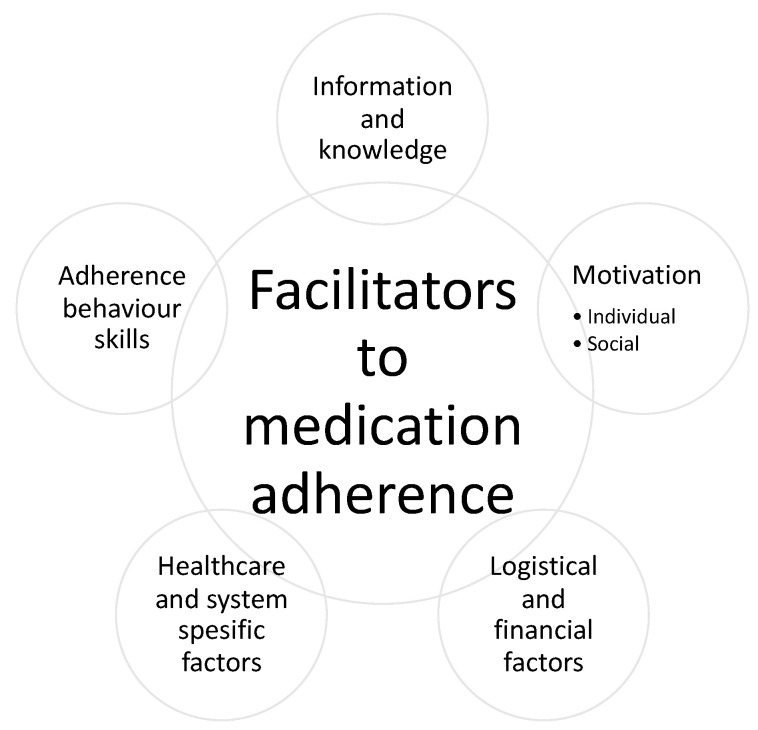
The identified key facilitators to medication adherence based on the included qualitative studies (*n* = 89).

**Figure 5:**
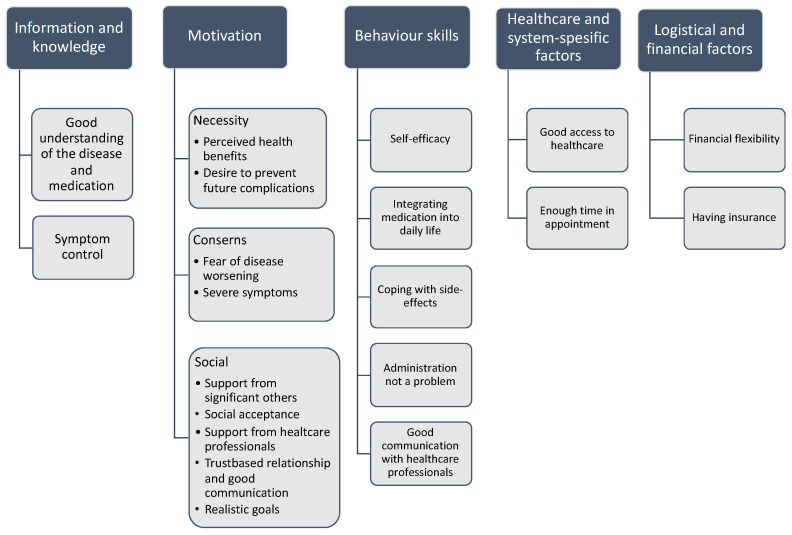
Categories and subcategories of facilitators to medication adherence arising from the included qualitative studies (*n* = 89). We used the Information–Motivation–Behaviour skills (IMB) model as part of the classification.

We identified five subject areas related to facilitators of medication adherence. Because medication taking is related to individual behaviour, we used the Information–Motivation–Behavioural Skills (IMB) Model as a starting point for the analysis [15,16]. However, as medication adherence is a complex entity in addition to human behaviour, we observed healthcare and system-specific factors and logistical and financial factors (Figure 4 and Figure 5).

#### 3.2.1. Informational, Motivational and Behavioural Factors

A good understanding of the illness and its treatment and how medicines promote the quality of life is essential for adherence [68]. The ability to integrate medications into daily life improves adherence in self-managing chronic conditions [79]. Low toxicity, mild adverse effects and an oral route of the administration seem to promote medication adherence [66,79]. There are different tools to assist with medicine taking, such as pillboxes, clock or mobile alarms, or taking medications during regular TV and radio programs [49,55].

The patient’s motivation is an essential facilitator. Motivation improves if the patient understands the necessity of the medication, and it contributes to positive health benefits [80]. Significant life events can have a positive effect on medication adherence. If a serious complication occurs, the importance of preventing complications and maintaining health is highlighted and may lead to a re-evaluation of the patient’s priorities [35,64,81]. The desire to return to “normal life” is a powerful facilitator to medication adherence [53].

The concerns related to illness may improve adherence and motivation to take medication as prescribed [79,82]. If patients have lived through the experience of their disease and its further negative impact on functional abilities, medication adherence may increase [82]. Knowing that interrupting or changing medications would result in the disease worsening can increase the desire to self-manage medication better [79]. The treatment goals must be realistic and achievable for the patient [52].

Support from family and friends and colleagues at work support adherence. It may require the disclosure of the illness, which can be scary for the patient [60,71]. Social acceptance helps the patient to cope with the illness.

Self-efficacy is an essential skill when coping with practical problems in daily life. If the patient takes ownership of self-managing the medication and knows how to adjust medicines if the disease worsens, the chances for better adherence are higher [60,83]. Feeling responsible and having a strong belief in the efficacy of medication promote self-empowerment and create a positive attitude towards the medication [59].

#### 3.2.2. Healthcare and System-Specific Facilitators

A trust-based, collaborative and respectful patient-provider relationship is crucial for medication adherence [55]. Good access to healthcare and enough time for discussions are necessary for patients [57]. Sometimes a desire to please healthcare providers or fearing them may also facilitate adherence [55]. Patients wish for confidential communication and an ongoing dialogue with health care professionals [84]. Support from healthcare providers and freely accessible care appear to increase adherence [65].

#### 3.2.3. Logistical and Financial Factors

Financial flexibility is necessary for medication adherence. The balance between revenue and expenditure of the household makes it possible to buy essential commodities such as food, clothes, and medicines without prioritising [80]. Additionally, having good insurance coverage guarantees secure finances in contrast to having no insurance at all.

### 3.3. Summary of Findings

We identified an extensive range of barriers and facilitators to medication adherence and the studies were more often concerned with barriers than facilitators. We classified the barriers as patient-specific, illness-specific, medication-related, healthcare and system-related, sociocultural and logistical and financial factors. The facilitators we identified were information and knowledge of the disease and medication, individual and social motivation, adherence behaviour skills, healthcare and system-specific factors and logistical and financial factors. Some of these factors can act as barriers and facilitators, such as healthcare and system-related factors and logistical and financial factors.

We identified similar factors to medication adherence in the previous systematic qualitative reviews (*n* = 13, Table A2) as in the qualitative studies described above (*n* = 76, Table A1). The previous systematic review findings confirm our own findings and the complexity of medication adherence as a phenomenon.

Some of the included studies had a theory-based approach to medication adherence (*n* = 17) (Table 1). Using different theories helped to understand and explain patients’ actions related to taking their medication (Table 1).

## 4. Discussion

### 4.1. Main Findings

To improve medication adherence, better communication and better information on the disease and its medication appeared to be the crucial concepts for patients in this scoping review. Our findings confirm that medication adherence is a complex phenomenon that is only partly understood. A wide range of factors seems to influence this either positively or negatively or in both ways. Regardless of the study concept, our findings were similar from study to study. Patients have many concerns about their illness, and it seems that they do not commonly have enough information to make knowledge-based decisions for self-managing their care. Patients want to discuss their problems and fears with a healthcare provider, but there is often not enough time for that in a short appointment.

According to this scoping review, the illness was not always a priority for the person. There can be many other matters in life that people prioritise more than their own optimal disease self-management. For better medication adherence, healthcare providers need to pay more attention to patients’ thoughts and concerns and have more time to listen to their experience in relation to the disease. Patients highly value trust-based relationships with healthcare providers.

This scoping review tracked many barriers that can hinder patients’ intention to adhere to their medication taking. The complexity of the matter may explain why many interventions to improve medication adherence are not successful [3]. If the intervention targets only some of the barriers, positive outcomes may be lacking, despite good intentions. The adherence to one medicine does not either automatically mean adherence to other medicines. Thus, adherence can differ from treatment to treatment or from disease to disease [68]. Patients may make their own priorities about the medications they use. This phenomenon should be further researched.

Our review of qualitative studies indicates that more attention should be paid to the patients’ fear of side effects. This can be a barrier that affects medication taking and can lead to skipping doses. With good knowledge and open and trust-based discussion with a healthcare provider, the patient need not begin to doubt their treatment. It is also good to discuss the patient’s values and religious values. A well-informed patient should know how to adjust medication to fit with religious requirements. The better the healthcare providers know the reality of their patients; living situations, the better they can support their patients to become empowered to self-manage a complex medication regimen.

Barriers can exist that the healthcare provider has not taken into consideration. The patient may have obstacles to self-manage taking their medication, for example, the difficulties of injecting medicines, remembering to take their medication on time when working, or the fear of stigmatisation. Additionally, financial obstacles can be difficult to reveal. Ideally, health care professionals should meet the patient without any preconceptions and in a trusted environment to discuss the barriers and concerns related to medication.

A theory-based approach may help to understand the patient’s actions and behaviours. However, a minority of the research we found had a theoretical approach, and the theories applied varied. Different behavioural theories, also adherence-specific ones, aim to explain chronically ill patient’s behaviour and give a reasonable explanation of why the patients act as they do. According to those theories, the patient’s action depends on their behaviour. This, in turn, depends on the patient’s beliefs or expected outcomes. According to our findings, an IMB model explains factors influencing adherence related to patient’s behaviour. However, external circumstances affect adherence, such as financial problems or poor access to care, which have to be considered. Based on our analyses, the different behavioural theories are good tools but do not fully explain complex adherence behaviour.

There is a need to generate new theory-based approaches to medication adherence since the current behavioural theories are not completely successful in explaining the complex phenomenon of adherence. There are also numerous adherence scales, which are very diverse and difficult to compare, so the research may need to be focused on comparing existing scales and determining which are most reliable. Qualitative research provides new insights into patient experiences and daily life struggles with their diseases and medicine taking to be incorporated in further development of the adherence measures and models. A good example of such a novel and promising conceptual approach is a model of medication-related burden and patients’ lived experiences with medicine, which builds on a meta-synthesis of qualitative studies [12].

Our review covered 13 systematic reviews, of which 11 were disease-specific, and 2 were generic. The illness-specific systematic reviews pointed out that patients often had misinterpretations of their illness, which prevented their adherence to medication. Clarifying these issues, having time and support, including from family members, were key recommendations to improve adherence in these reviews.

In our findings, there were more studies on barriers to medication adherence than facilitators. This is an interesting finding and can be affected by the fact that medication adherence is poorly understood, at least how it can be improved. When trying to understand the patient’s struggle with complex medication regimens, it may have caused the focus of research to go more towards barriers than facilitators. This scoping review may help to better understand the broader picture of adherence and to find interventions and strategies to improve it.

To our knowledge, this is the first scoping review on patient-related factors of medication adherence based on qualitative research. We conclude that well-informed patients and trustful patient-provider relationships are at the centre of improving medication adherence. Self-efficacy is crucial and empowers the patient to control and self-manage the disease and adjust the medication when necessary. Patient motivation needs to be monitored and supported. Moreover, patients need help integrating the medication regimen into their daily lives and to have routines. Support from significant others is essential too. They can support the patient in a life-long journey with the disease and give motivation for good medication self-management.

However, more research is needed to understand the patient’s reality. This scoping review clarifies the contributing factors of nonadherence and why the outcomes of interventions to improve adherence can be poor. The observations presented in this scoping review are useful when planning more effective interventions to increase medication adherence.

### 4.2. Strengths and Limitations

This scoping review of qualitative studies provides new information on people’s medicine-taking behaviour, which may not have been used to the best advantage. The strengths of this scoping review include an extensive literature search and review, followed by a thorough categorization of the barriers and facilitators of medication adherence. The literature searches were made with the support of an experienced librarian, and we had good coverage of qualitative studies where the primary focus was patients’ experiences and attitudes towards medication adherence. To avoid a selection bias, there were three researchers involved in the selection process. The data was thoroughly extracted and analysed to define the overarching categories.

A limitation is that since we focused on the qualitative aspects, we cannot conclude the magnitude of the effect of several factors influencing adherence. We also limited our search to studies in English, which may be a source of bias. The studies reported more barriers than facilitators, which may be another limitation. On the other hand, it describes the fact that barriers have been better recognized than facilitators. More research should be focused on the factors that have been able to help patients to commit to their disease and medication self-management. More research is also needed to elaborate on new theoretical models. This scoping review provides a good basis for building up more comprehensive theoretical models on medication adherence.

## 5. Conclusions

This scoping review highlighted a wide range of barriers and facilitators. The barriers seem to be better known than the facilitators. There is a need for better recognition of facilitators. We may need to increase the qualitative research of medication adherence to better understand the patients lived experiences that direct their medicine-taking behaviour. This information is needed to find new interventions and approaches to increase medication adherence, compare existing adherence scales, and build up more comprehensive theoretical models on medication adherence.

Patients wish to discuss their concerns about medications. Better communication and information appear to be among the most crucial factors for patients. The factors presented in this scoping review may help clinicians who communicate with patients having issues with adherence. The findings of this scoping review may also help those who plan further interventions to build up a more comprehensive approach to improve medication adherence.

## Figures and Tables

**Table 1:** Summary of the theories used in the included studies.

Theory	Medication Therapy	Study
**ABC Taxonomy and Three Factor Model**	Hypertension, heart disease, COPD, asthma	Maffoni et al., 2020
**Andersen’s Behavioural Model**	Antiretroviral therapy Antiretroviral therapy	Holtzman et al., 2015 Schatz et al., 2019
**Common-Sense Model of Self-Regulation (CSM)**	Glaucoma medication	McDonald et al., 2019
**Dowell’s Therapeutic Alliance**	Cardio-protective medication	Lambert-Kerzner et al., 2015
**Dowell’s Therapeutic Alliance Model and Leventhal’s Common-Sense Model**	Use of prescription medicines in general	Kucukarslan et al., 2012
**Health Belief Model**	Heart medication Clopidogrel Rheumatoid arthritis Hypertension	Garavalia et al., 2009 Garavalia et.al., 2011 Oshotse et.al., 2018 Rahmawati et al., 2018
**Health Literacy Pathway Model**	Diabetes type 2 medication	Huang et al., 2020
**Information-Motivation-Behaviour Skills (IMB) Model of Adherence**	Chronic hepatitis C therapy	Evon et al., 2015
**Naturalistic Decision-making Model**	Heart failure	Meraz et.al., 2020
**Roy Adaptation Model**	Diabetes type 2 medication	Bockwold et al., 2017
**Social Ecological Model**	Cardiovascular medication Antiretrovial therapy	Petterssen et al., 2018 Becker et al., 2020
**Stages of Change Model**	Anti-diabetic medication	Sapkota et al., 2018

## Data Availability

The available data is included in Appendix A, Appendix B and Appendix C. Other data used in this study are available on request from the corresponding author.

## Appendix A

Search Strategy for the Scopus Database

(TITLE-ABS-KEY ((medication * OR drug * OR medicine *) W/2 (adhere * OR non-adheren * OR nonadheren * OR complian * OR noncomplian *))) AND (TITLE-ABS-KEY (patient * W/2 (experienc * OR fear * OR belie * OR knowled * OR attitude OR behavio * OR communicat * OR reason OR reasons OR cause *))) AND (TITLE-ABS-KEY (qualitative OR interview * OR “focus group *” OR questionnaire * OR (observation * W/2 (study OR research)))) AND (LIMIT-TO (PUBYEAR, 2019) OR LIMIT-TO (PUBYEAR, 2018) OR LIMIT-TO (PUBYEAR, 2017) OR LIMIT-TO (PUBYEAR, 2016) OR LIMIT-TO (PUBYEAR, 2015) OR LIMIT-TO (PUBYEAR, 2014) OR LIMIT-TO (PUBYEAR, 2013) OR LIMIT-TO (PUBYEAR, 2012) OR LIMIT-TO (PUBYEAR, 2011) OR LIMIT-TO (PUBYEAR, 2010) OR LIMIT-TO (PUBYEAR, 2009))

## Appendix B

**Table A1:** Summary of included studies.

Study	Title	Study Design	Concept	Context	Illness	Country in Which the Study Conducted
Al-Qazaz et al., 2011	Perception and knowledge of patients with type 2diabetes in Malaysia about their disease and medication: A qualitative study	Individual interviews	Diabetic’ patients’ experience and knowledge about diabetes and its medication and the factors contributing to medication adherence	Diabetic 2 patients’ adherence to medication and knowledge about their illness	Cardiovascular disease	Malaysia
AlHamid et al., 2014	A systematic review of qualitative research on the contributory factors leading to medicine-related problems from the perspectives of adult patients with cardiovascular diseases and diabetes mellitus	Systematic review	To explore and evaluate contributory factors leading to MRPs among adult patients with CVDs and/or DM from their perspectives	Outpatients with diabetes or cardiovascular diseases	DM and cardiovascular disease	12 countries: Australia, Brazil, Cameroon, Canada, Croatia, Ireland, Malaysia, South Africa, Spain, Taiwan, the UK, and the USA
Ali et al., 2019	Qualitative Analysis of Factors Influencing Patient Persistence and Adherence to Prescribed Overactive Bladder Medication in UK Primary Care	Individual interviews	Non-Adherence has a major impact on health outcomes in long term diseases.	Primary care in the UK	Overactive bladder	UK
Alodhaib et al., 2021	Qualitative Exploration of Barriers to Medication Adherence Among Patients with Uncontrolled Diabetes in Saudi Arabia	Individual interviews	Physicians can rarely identify non-adherent patients	Patients at a diabetes centre in Saudi-Arabia	DM	Saudi-Arabia
Alhomoud et al., 2015	South Asian, and Middle Eastern patients’ perspectives on medicine-related problems in the United Kingdom	Individual interviews	Medication adherence	Outpatients in community pharmacies in London (*n* = 94)	Not mentioned (patients in general)	UK
Axelsson et al., 2015	Antiretroviral therapy adherence strategies used by patients of a large HIV clinic in Lesotho	Individual interviews	Adherence to ART (outpatients)	Patients receiving ART in their monthly clinical visit	HIV/AIDS	Lesotho
Barasa Masaba et al., 2020	Determinants of Non-Adherence to Treatment Among Patients with Type 2 Diabetes in Kenya: A Systematic Review. (00Review)	Systematic review	Diabetes is the leading non-communicable disease in Kenya	Health care in Kenya	DM2	Kenya
Becker et al., 2020	Individual, household, and community level barriers to ART adherence among women in rural Eswatini	Focus group	Barriers to ART among woman living with HIV in communities	Rural women living with HIV/AIDS	HIV/AIDS	Eswatini, Africa
Bezabhe et al., 2014	Barriers and Facilitators of Adherence to Antiretroviral Drug Therapy and Retention in Care among Adult HIV-Positive Patients: A Qualitative Study from Ethiopia	Individual interviews	Adherence to ART	Outpatients receiving ART in HIV-clinic, problems in medication taking	HIV/AIDS	Ethiopia
Bockwold et al., 2017	Understanding experiences of diabetes medications among African Americans living with type 2 diabetes	Individual interviews	The contributing factors to non-adherence to diabetic medications in AAs	Hospital-based outpatient diabetes clinic in low-income Chicago	DM2	United States
Chen et al., 2014	Disease acceptance and adherence to imatinib in Taiwanese chronic myeloid leukaemia outpatients	Individual interviews	Adherence to imatinib, a medication for CML	Outpatient clinic in Taiwan	Chronic myeloid leukaemia	Taiwan
Clancy et al., 2020	Breast cancer patients’ experiences of adherence and persistence to oral endocrine therapy: A qualitative evidence synthesis	Systematic review	Breast cancer patients’ experiences of adherence	Outpatient setting	Breast cancer	Ireland
Dehdari et al., 2019	The determinants of anti-diabetic medication adherence based on the experiences of type 2 diabetes	Individual interviews	Medication adherence as presented by type 2 DM patients and their families	Secondary care outpatient clinic in Iran	DM2	Iran
Eliasson et al., 2011	Exploring chronic myeloid leukaemia patients’ reasons for not adhering to the oral anticancer drug imatinib as prescribed	Individual interviews	CML outpatients who have been prescribed imatinib	CML outpatients with prescribed imatinib medication	Chronic myeloid leukaemia	United States
Evon et al., 2015	Adherence during Antiviral Treatment Regimens for Chronic Hepatitis C: A Qualitative Study of Patient-Reported Facilitators and Barriers	Individual interviews	HCV patients’ adherence taking HCV medication as prescribed	Outpatients who receive HCV medication	Chronic Hepatitis C	United States
Farinha et al., 2017	Concerns of patients with systemic lupus erythematosus and adherence to therapy—a qualitative study	Individual interviews	SLE outpatients thoughts and concerns about their illness and medication management	Outpatient clinics	Systemic lupus erythematosus	Portugal
Frech et al., 2021	Patterns and facilitators for the promotion of glaucoma medication adherence-a qualitative study	Individual interviews	Better understanding of patient patterns in glaucoma medication management	Department of Ophthalmology in Germany	Glaucoma	Germany
Garavalia et al., 2009	Exploring Patients’ Reasons for Discontinuance of Heart Medications	Individual interviews	To understand patients’ viewpoints on why they stopped taking their medication	MI outpatients who have stopped to take their prescribed medication (clopidogrel or cholesterol lowering medication)	Cardiovascular disease	United States
Garavalia et al., 2011	Clinician-Patient Discord: Exploring Differences in Perspectives for Discontinuing Clopidogrel	Individual interviews	Why MI outpatients stop to take clopidogrel	Outpatients setting	Cardiovascular disease	United States
Gassmann et al., 2016	Experiences and coping strategies of oncology patients undergoing oral chemotherapy: First steps of a grounded theory study	Individual interviews	Patients’ thoughts about their oral chemotherapy management	Outpatients receiving oral chemotherapy	Oncology patients	Switzerland
Goldsmith et al., 2017	Understanding the patient experience of cost-related non-adherence to prescription medications through typology development and application	Individual interviews	In which situation patients make the decision not to purchase medicines	Outpatients who have not taken their drugs as prescribed because of cost-related problems	Patients who have experiences cost-related-non-adherence	Canada
Habte et al., 2017	Barriers and facilitators to adherence to anti-diabetic medications: Ethiopian patients’ perspectives	Individual interviews	Patients’ anti-diabetic medication-taking experiences	Diabetes 2 patients in public outpatient clinics	DM2	Ethiopia
Harrold et al., 2010	Patients and providers view gout differently: a qualitative study	Individual interviews	Gout patients views to ULD (urate lowering drugs)	Gout patients’ adherence to long-term urate lowering medication	Gout	United States
Hayden et al., 2015	Patients’ adherence-related beliefs about methotrexate: a qualitative study of the role of written patient information	Individual interviews	How patients’ beliefs and concerns about methotrexate affected their medicine taking	Patients’ decisions about taking methotrexate in outpatients setting	Inflammatory arthritis	UK
Hedenrud et al., 2019	“I did not know it was so important to take it the whole time”—self-reported barriers to medical treatment among individuals with asthma	Individual interviews	Explore self-perceived barriers to medication adherence	Outpatient setting	Asthma	Sweden
Ho et al., 2017	Barriers and facilitators of adherence to antidepressants among outpatients with major depressive disorder: A qualitative study	Individual interviews	Patients’ non-adherence in depression medication	Psychiatric clinic in government-run hospital in Malaysia	Depression	Malaysia
Hogan et al., 2014	Factors affecting nebulised medicine adherence in adult patients with cystic fibrosis: a qualitative study	Individual interviews	Barriers and facilitators of nebulised medicines used for cystic fibrosis	Patients recruited through a patient organisation	Cystic fibrosis	Australia
Holtzman et al., 2015	Mapping patient-identified barriers and facilitators to retention in HIV care and antiretroviral therapy adherence to Andersen’s behavioural model	Individual interviews	HIV medication adherence linked to ABM model	HIV clinics in Philadelphia, USA	HIV/AIDS	United States
Huang et al., 2020	“Why Am I Not Taking Medications?” Barriers and Facilitators of Diabetes Medication Adherence Across Different Health Literacy Levels	Individual interviews	To explore patients’ perceptions of the barriers to and facilitators of medication adherence across different levels of health literacy	how individuals make decisions within an actual real-world situation	DM2	United States
Iacorossi et al., 2019	Qualitative study of patients with metastatic prostate cancer to adherence of hormone therapy	Individual interviews	Adherence to oral hormone treatment	Outpatient setting	Cancer	Italy
Jaffray et al., 2014	Why do patients discontinue antidepressant therapy early? A qualitative study	Individual interviews	Factors that hinder or facilitate the continuation of AD therapy	Patients treated in general practice in Scotland	Depression	UK
Jarab et al., 2018	A focus group study of patient’s perspective and experiences of type 2 diabetes and its management in Jordan	Focus group	Non-adherence to medication as major barrier to achieve good results in diabetes care	Hospital outpatients in Jordan	DM2	Jordan
Jeragh-Alhaddad et al., 2015	Barriers to medication taking among Kuwaiti patients with type 2 diabetes: A qualitative study	Individual interviews	Non-adherence to medication remains an unresolved problem	Type 2 DM patients from general practice and hospitals	DM2	Kuwait
Ju et al., 2018	Patient beliefs and attitudes to taking statins: Systematic review of qualitative studies	Systematic review	Patients’ perspective on statins in primary or secondary prevention of CVD	Systematic review on qualitative studies on patients with statins	Cardiovascular disease	Australia
Kassavou et al., 2017	Reasons for non-adherence to cardiometabolic medications, and acceptability of an interactive voice response intervention in patients with hypertension and type 2 diabetes in primary care: a qualitative study	Individual interviews	Patients’ non-adherence to cardio metabolic medications	General practice patients in the UK	DM2 and/or hypertension	UK
Kelly et al., 2014	Knowledge, attitudes, and beliefs of patients and carersregarding medication adherence: a review of qualitative literature	Systematic review	Systematic qualitative review on factors which can affect medication adherence	Outpatients setting	Not mentioned (patients in general)	Ireland
Kelly et al., 2018	Patients’ Attitudes and Experiences of Disease-Modifying Antirheumatic Drugs in Rheumatoid Arthritis and Spondylarthritis: A Qualitative Synthesis	Systematic review	Non-adherence to antirheumatic drugs	Qualitative studies on adherence to RA drugs	Rheumatoid arthritis and spondylarthritis	Australia
King-Shier et al., 2017	Ethno-Cultural Considerations in Cardiac Patients’ Medication Adherence	Individual interviews	How patients manage to take their cardiac medication	Outpatient setting	Cardiovascular disease	Canada
Kobue et al., 2017	“It’s so hard taking pills when you don’t know what they’re for”: a qualitative study of patients’ medicine taking behaviours and conceptualisation of medicines in the context of rheumatoid arthritis	Individual interviews	To understand RA patients medicine taking behaviour	Patients with Rheumatoid Arthritis (RA) living in South Africa	Rheumatoid arthritis	South Africa
Koh et al., 2018	Access and adherence to medications for the primary and secondary prevention of atherosclerotic cardiovascular disease in Singapore: a qualitative study	Individual interviews	To understand factors influencing patients’ adherence to treatment	Factors impacting on adherence with cardiovascular patients	Cardiovascular disease	Singapore
Kucukarslan et al., 2012	Exploring patient experiences with prescription medicines to identify unmet patient needs: Implications for research and practice	Focus group	Patients’ unmet needs when taking prescribed medicines	Outpatients in the US	Cardiovascular disease	United States
Lambert-Kerzner et al., 2015	Perspectives of patients on factors relating to adherence to post-acute coronary syndrome medical regimens	Individual interviews	Adherence to cardio protective medications after acute coronary syndrome	Patients attending an RCT on multi-faceted intervention to improve cardiac medication adherence	Cardiovascular disease	United States
Lyimo et al., 2012	Determinants of antiretroviral therapy adherence in northern Tanzania: a comprehensive picture from the patient perspective	Individual interviews	Understanding of barriers and facilitators of antiretroviral therapy	Health centres in Tanzania	HIV/AIDS	Tanzania
Maffoni et al., 2020	Medication adherence in the older adults with chronic multimorbidity: a systematic review of qualitative studies on patient’s experience. [Review]	Systematic review	Older patient’s perspective on medication adherence	medication adherence in chronic diseases	Other: hypertension, heart disease, COPD, asthma	Italy, Portugal, and Poland
Marshall et al., 2012	Lay perspectives on hypertension and drug adherence: systematic review of qualitative research	Systematic review	To better understand patients’ perspectives to medication adherence	Qualitative studies on patients using antihypertensive drugs	Cardiovascular disease	UK
McDonald et al., 2019	A theory-driven qualitative study exploring issues relating to adherence to topical glaucoma medications	Individual interviews	Investigating patients’ perceptions of their illness	Two outpatient glaucoma clinics	Glaucoma	UK
McKillop et al., 2013	Patients’ experience and perceptions of polypharmacy in chronic kidney disease and its impact on adherent behaviour	Individual interviews	Polypharmacy is common in chronic kidney disease and associated with medication adherence	Patients at a nephrology clinic	Chronic kidney disease	UK
McSharry et al., 2016	Systematic Review or Meta-analysis Perceptions and experiences of taking oral medications for the treatment of Type 2 diabetes mellitus: a systematic review and meta-synthesis of qualitative studies	Systematic review	DM2 patients’ adherence to diabetes medicines	Outpatients setting	DM2	UK
Meraz et al., 2020	Medication Non-adherence or Self-care? Understanding the Medication Decision-Making Process and Experiences of Older Adults With Heart Failure	Individual interviews	Understanding the medicine decision-making process	Community setting	Cardiovascular disease	United States
Miller et al., 2010	Why are antiretroviral treatment patients lost to follow-up? A qualitative study from South Africa	Individual interviews	Reasons for non-adherence	HIV/AIDS patients receiving ART	HIV/AIDS	South Africa
Ming et al., 2011	Perspectives of heart failure patients in Malaysia towards medications and disease state management: Findings from a qualitative study	Individual interviews	Patient perspectives in the management of heart failure	General hospital, Malaysia	Cardiovascular disease	Malaysia
Mostafavi et al., 2021	The psychosocial barriers to medication adherence of patients with type 2 diabetes: a qualitative study	Individual interviews	Barriers to medication adherence	Outpatient setting	DM2	Iran
Muiruri et al., 2020	Why do people living with HIV adhere to antiretroviral therapy and not comorbid cardiovascular disease medications? A qualitative inquiry	Focus groups and individual interviews	HIV-patients adherence to cardiovascular medications	Outpatient setting	Cardiovascular disease	United States
Nielsen et al., 2018	Adherence to medication in patients with chronic kidney disease: a systematic review of qualitative research	Systematic review	Non-adherence to multipharmacological treatment	Nephrology unit in Denmark	Chronic kidney disease	Denmark
Oshotse et al., 2018	Self-Efficacy and Adherence Behaviours in Rheumatoid Arthritis Patients	Other: Focus group and individual interviews	How self-efficacy and adherence is influencing medication taking	RA patients’ self-management	Rheumatoid arthritis	United States
Pagès-Puigdemont et al., 2016	Patients’ Perspective of Medication Adherence in Chronic Conditions: A Qualitative Study	Focus group	Medication adherence in chronic conditions	Patents’ perspectives in medication management in chronic diseases	Chronic diseases	Spain
Patel et al., 2015	Concerns and perceptions about necessity in relation to insulin therapy in an ethnically diverse UK population with type 2 diabetes: a qualitative study focusing mainly on people of South Asian origin	Individual interviews	Accepting insulin as medication to DM2	Ethnic population living in UK with DM2 and their adherence to insulin	DM2	UK
Peláez et al., 2016	How can adherence to asthma medication been enhanced? Triangulation of key asthma stakeholders’ perspectives	Focus group	Asthma patients’ adherence to medications	To explore interventions which enhances adherence to asthma medication in long-term	Asthma	Canada
Pettersen et al., 2018	Challenges adhering to a medication regimen following first-time percutaneous coronary intervention: A patient perspective	Individual interviews	Adherence to cardiovascular medication after percutaneous coronary intervention	Cardiology unit in Norway	Cardiovascular disease	Norway
Polinski et al., 2014	A matter of trust: patient barriers to primary medication adherence	Focus group	Patients’ adherence to antihypertensive medications remains suboptimal	Patients who did not pick up the first antihypertensive prescription	Cardiovascular disease	United States
Rahmawati et al., 2018	Understanding untreated hypertension from patients’ point of view: A qualitative study in rural Yogyakarta province, Indonesia	Individual interviews	To explore perspectives from patients who do not take anti-hypertensive medications		Cardiovascular disease	Indonesia
Rashid et al., 2014	Medication taking in coronary artery disease: A systematic review and qualitative synthesis	Systematic review	Patients’ discontinuation to secondary prevention medication for coronary artery disease	Qualitative research about the medication-taking experiences	Cardiovascular disease	UK
Rathbone et al., 2017	A systematic review and thematic synthesis of patients’ experience of medicines adherence	Systematic review	Phenomenology has a place in studying adherence	Phenomenological papers studying medication adherence	Cardiovascular disease	UK
Rezaei et al., 2019	Barriers of medication adherence in patients with type-2 diabetes: A pilot qualitative study	Individual interviews	Patients with type 2 diabetes have poor adherence to the therapeutic regime	Outpatient setting	DM2	Iran
Richardson et al., 2016	A joint effort over a period of time: Factors affecting use of urate-lowering therapy for long-Term treatment of gout	Individual interviews	Reasons for non-adherence to gout treatment	GP patients through the UK	Gout	UK
Rifkin et al., 2010	Medication adherence behaviour and priorities among older adults with CKD: A semistructured interview study	Individual interviews	How patients with multiple problems in kidney disease prioritise their medications	Community-dwelling patients with kidney disease	Chronic kidney disease	United States
Rowell-Cunsolo et al., 2020	Barriers to optimal antiretroviral therapy adherence among HIV-infected formerly incarcerated individuals in New York City	Individual interviews	Investigate barriers to ART	Outpatient setting	HIV/AIDS	United States
Saleem et al., 2012	Drug attitude and adherence: A qualitative insight of patients with hypertension	Individual interviews	Patients’ insight to hypertension medication	Outpatient setting	Cardiovascular disease	Pakistan
Sapkota et al., 2018	Nepalese patients’ anti-diabetic medication taking behaviour: an exploratory study	Individual interviews	Diabetes causes a huge burden for low- and middle-income countries	Nepalese type 2 DM patients in Nepal and Australia	DM2	Nepal and Australia
Schatz et al., 2019	“For us here, we remind ourselves”: strategies and barriers to ART access and adherence among older Ugandans	Individual interviews	Antiretroviral therapy among older Africans	Older adults in Uganda with HIV	HIV/AIDS	Uganda
Shalihu et al., 2014	Namibian prisoners describe barriers to HIV antiretroviral therapy adherence	Individual interviews	Adherence to HIV medication	Patients with AIDS in a Namibian prison	HIV/AIDS	Namibia
Shaw et al., 2018	Rheumatoid arthritis patients’ motivations for accepting or resisting disease-modifying antirheumatic drug treatment regimens	Individual interviews	Patients’ decision to accept or resist disease modifying anti rheumatic drugs	Four rheumatology clinics in Pittsburgh	Rheumatoid arthritis	United States
Shiyanbola et al., 2018	“I did not want to take that medicine”: African-Americans’ reasons for diabetes medication non-adherence and perceived solutions for enhancing adherence	Focus group	Diabetes is disproportionally burdensome among African-Americans (AAs) and medication adherence is important for optimal outcomes.	Community African American type 2 DM patients	DM2	United States
Souter et al., 2014	Optimisation of secondary prevention of stroke: A qualitative study of stroke patients’ beliefs, concerns and difficulties with their medicines	Individual interviews	Optimisation of secondary prevention of stroke	Patients discharged from stroke rehabilitation	Cardiovascular disease	UK
Srimongkon et al., 2018	Consumer-related factors influencing antidepressant adherence in unipolar depression: a qualitative study	Individual interviews	Adherence at different stages: initiation, implementation, and discontinuation of medication	Outpatient setting	Depression	Australia
Stern et al., 2017	Conceptions of agency and constraint for HIV-positive patients and healthcare workers to support long-term engagement with antiretroviral therapy care in Khayelitsha, South Africa	Individual interviews	Barriers to long-term ART adherence is critical in HIV management	Three HIV clinics in South Africa	HIV/AIDS	South Africa
Stryker et al., 2010	An Exploratory Study of Factors Influencing Glaucoma Treatment Adherence	Individual interviews	Patient adherence to glaucoma treatment regimens is often suboptimal	Veteran Affairs hospital in US	Glaucoma	United States
Tong et al., 2011	The perspectives of kidney transplant recipients on medicine taking: A systematic review of qualitative studies	Systematic review	Non-adherence to medication regimens after kidney transplantation	Qualitative studies using interviews, focus groups, document analysis or observations to explore the perspectives of adult kidney transplant recipients	Kidney transplants	Australia
Tordoff et al, 2010	‘‘It’s just routine.’’ A qualitative study of medicine-taking amongst older people in New Zealand	Individual interviews	Many older people use large numbers of medicines and are more likely to have problems taking them	Community patients in New Zealand	Not mentioned (patients in general)	New Zealand
Tranulis et al., 2011	Becoming adherent to antipsychotics: a qualitative study of treatment-experienced schizophrenia patients	Individual interviews	Patients’ perspectives on the discontinuation of antipsychotics	Community mental health centre outpatient clinic	Schizophrenia	United States
Vaanholt et al., 2018	Perceived advantages and disadvantages of oral anticoagulants, and the trade-offs patients make in choosing anticoagulant therapy and adhering to their drug regimen	Focus group	Adherence to oral anticoagulants	AF patients in different European countries	Cardiovascular disease	Netherland
Van Geffen et al., 2011	The decision to continue or discontinue treatment: Experiences and beliefs of users of selective serotonin-reuptake inhibitors in the initial months—A qualitative study	Individual interviews	To identify what reasons lead to discontinuation or continuation of treatment	Depression patients in community pharmacies 3 months after starting SSRI treatment	Depression	Netherland
Van Tam et al., 2011	“It is not that I forget, it’s just that I don’t want other people to know”: barriers to and strategies for adherence to antiretroviral therapy among HIV patients in Northern Vietnam	Focus group	Little is known about factors influencing ART adherence among people living with HIV	HIV patients in Vietnam	HIV/AIDS	Vietnam
VanLoggerenberg et al., 2015	A Qualitative Study of Patient Motivation to Adhere to Combination Antiretroviral Therapy in South Africa	Other: Individual interviews and focus groups	Adherence to ART	Patients receiving ART medication at the clinic	HIV/AIDS	South Africa
Verbrugghe et al., 2016	Factors influencing adherence in cancer patients taking oral tyrosine kinase inhibitors	Individual interviews	Non-adherence in cancer patients taking oral anticancer drugs is common	Five hospitals in Belgium	Cancer	Belgium
Vipey et al., 2021	A qualitative study of barriers and facilitators to adherence to secondary prevention medications among French patients suffering from stroke and transient ischemic attack	Individual interviews	TIA patients do not adhere to their secondary prevention medicines	Cohort of TIA patients in France	Cardiovascular disease	France
Widayanti et al., 2020	Medicine taking behaviours of people with type 2 diabetes in Indonesia: a qualitative study	Focus group	Rural and urban communities	People’s medicine-taking behaviours	DM	Indonesia
Wu et al., 2015	Lack of congruence between patients’ and health professionals’ perspectives of adherence to imatinib therapy in treatment of chronic myeloid leukaemia: A qualitative study	Individual interviews	Consistent use of imatinib is critical for treatment success in chronic myeloid leukaemia	Patients in specialised cancer centres (health professionals too)	Chronic myeloid leukaemia	Australia
Ågärd et al., 2016	Diabetes in the shadow of daily life: factors that make diabetes a marginal problem	Individual interviews	Diabetic patients’ challenges in following treatment recommendations	Medical outpatient clinic in Sweden	DM2	Sweden

## Appendix C

**Table A2:** Summary of systematic reviews.

Study	Title	Country in Which the Study Conducted	Aim of Study	Study Design	Data Sources	Results According to the Research Articles	Population Description	Total Number of Participants
Al Hamid et al., 2014	A systematic review of qualitative research on the contributory factors leading to medicine-related problems from the perspectives of adult patients with cardiovascular diseases and diabetes mellitus	12 countries: Australia, Brazil, Cameroon, Canada, Croatia, Ireland, Malaysia, South Africa, Spain, Taiwan, the UK and the USA	To explore and evaluate contributory factors leading to MRPs among adult patients with CVDs and/or DM from their perspectives.	Systematic review	Pubmed, EMBASE, ISIWeb of Knowledge, PsycInfo, International Pharmaceutical Abstract and PsycExtra	Patient-related factors including socioeconomic factors (beliefs, feeling victimised, history of the condition, lack of finance, lack of motivation, and low self-esteem) and lifestyle factors (diet, lack of exercise/time to see the doctor, obesity, smoking, and stress), medicine-related factors (belief in natural remedies, fear of medicine, lack of belief in medicines, lack of knowledge, non- adherence, and polypharmacy) and condition-related factors (lack of knowledge/understanding, fear of condition and its complications, and lack of control).	Adult patients with cardiovascular disease and/or diabetes	836 (21 studies)
Barasa Masaba et al., 2020	Determinants of Non-Adherence to Treatment Among Patients with Type 2 Diabetes in Kenya: A Systematic Review. [Review]	Kenya	What are the determinants that contribute to non-adherence to treatment among patients with T2DM in Kenya	Systematic review	Scopus, Web of Science, Science Direct, Cochrane Library, PUBMED, OVID and Google Scholar.	(1) Cost—income, insurance, distance, bills of drugs and food; (2) Patient characteristics—perception of (efficacy, severity, effects of non-adherence), knowledge, co-morbidity, family support, self-unfounded beliefs; and (3) Health system—health education, multiple drugs, evaluations and support, guidelines, and poor perception of system.	Adult patients with type 2 diabetes	15 studies
Clancy et al., 2020	Breast cancer patients’ experiences of adherence and persistence to oral endocrine therapy: A qualitative evidence synthesis	Ireland	To synthesise breast cancer patients’ experiences of adherence and persistence to oral endocrine therapy.	Systematic review	Embase, Cinahl, Pubmed, Psychinfo, Proquest, Lenus, Scopus, Web of Science, Rian.ie, EThOS e-theses online, DART Europe. No year limit was set	Three analytic themes were identified (We do not have an option; the side effects are worse than the disease; help us with information and support). Non-adherence and non-persistence were associated with debilitating side effects, inadequate information, and lack of support.	Breast cancer patients	42 studies
Ju et al., 2018	Patient beliefs and attitudes to taking statins: Systematic review of qualitative studies	Australia	To describe patients’ perspectives, experience, and attitudes towards statins	Systematic review	PsycINFO, CINAHL, Embase, MEDLINE, and PhD dissertations from inception to 6 October 2016	Confidence in prevention (trust in efficacy, minimising long-term catastrophic CVD, taking control, easing anxiety about high cholesterol); routinising into daily life; questioning utility (imperceptible benefits, uncertainties about pharmacological mechanisms); medical distrust (scepticism about overprescribing, pressure to start therapy); threatening health (competing priorities and risks, debilitating side effects, toxicity to body); signifying sickness (fear of perpetual dependence, losing the battle); and financial strain.	cardiovascular patients using statins in different countries	888 (32 studies)
Kelly et al., 2014	Knowledge, attitudes, and beliefs of patients and carers regarding medication adherence: a review of qualitative literature	Ireland	Knowledge, attitudes, and beliefs of patients and carers regarding medication adherence	Systematic review	CINAHL, Embase, PubMed, and Web of Knowledge from inception to November 2013.	Eight themes were identified: (1) beliefs and experiences of medicines, (2) family support and culture, (3) role of and relationship with healthcare practitioners, (4) factors related to the disease, (5) self-regulation, (6) communication, (7) cost, and (8) access.	Users of medicines (not mentioned, patients in general)	34 studies
Kelly et al., 2018	Patients’ Attitudes and Experiences of Disease-Modifying Antirheumatic Drugs in Rheumatoid Arthritis and Spondylarthritis: A Qualitative Synthesis	Australia	To describe patients’ attitudes and experiences of DMARDS in RA and spondylarthritis	Systematic review	MEDLINE, Embase, PsycINFO, and CINAHL were searched to January 2016	Six themes were identifyed with subthemes: intensifying disease identity (severity of sudden pharmacotherapy, signifying deteriorating health, daunting lifelong therapy), distressing uncertainties and consequences (poisoning the body, doubting efficacy, conflicting and confusing advice, prognostic uncertainty with changing treatment regimens), powerful social influences (swayed by others’ experiences, partnering with physicians, maintaining roles, confidence in comprehensive and ongoing care, valuing peer support), privilege and right of access to biologic agents (expensive medications must be better, right to receive a biologic agent, fearing dispossession), maintaining control (complete ownership of decision, taking extreme risks, minimizing life- style intrusion), and negotiating treatment expectations (miraculous recovery, mediocre benefit, reaching the end of the line).	Adults (ages ≥18 years) with rheumatoid arthritis or spondylarthritis	1383 (56 studies)
Maffoni et al., 2020	Medication adherence in the older adults with chronic multimorbidity: a systematic review of qualitative studies on patient’s experience	Italy, Portugal, Poland	To investigate potential factors associated with medication adherence in the older and chronic population through a PRISMA systematic review of qualitative studies on patients’ experience.	Systematic review	Scopus and Pubmed from 2000 to October 2017	According to the ABC Taxonomy, Persistence and Implementation were the most often considered phases. Considering the Three Factor model, the most often reported themes were Information and Strategies upon being adherent. The patient’s decisional flowchart describing barriers and facilitators (personal, social and environmental) to adherence was proposed.	Older adults aged 65 or more	39 studies
Marshall et al., 2012	Lay perspectives on hypertension and drug adherence: systematic review of qualitative research	UK	To examine lay understanding of hypertension and perspectives on drug taking	Systematic review	Medline, Embase, the British Nursing Index, Social Policy and Practice, and PsycInfo from inception to October 2011	A large proportion of participants thought hypertension was principally caused by stress and produced symptoms, particularly headache, dizziness, and sweating. Participants widely intentionally reduced or stopped treatment without consulting their doctor, commonly perceived that their blood pressure improved when symptoms abated or when they were not stressed, and that treatment was not needed at these times. Participants disliked treatment and its side effects and feared addiction. Participants reported various external factors that prevented adherence: being unable to find time to take the drugs or to see the doctor; having insufficient money to pay for treatment; the cost of appointments and healthy food; a lack of health insurance; and forgetfulness.	A global population with cardiovascular disease	53 qualitative studies
McSharry et al., 2016	Systematic Review or Meta-analysis Perceptions and experiences of taking oral medications for the treatment of Type 2 diabetes mellitus: a systematic review and meta-synthesis of qualitative studies	UK	To explore patients’ perceptions and experiences of taking oral medications for the pharmacological management of Type 2 diabetes by carrying out a systematic review and qualitative meta-synthesis of published qualitative studies	Systematic review	Cinahl, EMBASE, Medline, and PsycINFO databases were searched in 2014	Medications for diabetes: a necessary evil, outlines how patients’ negative perceptions of medication risks co-exist with a resounding view that medications are beneficial. Passive patients but active experimenters highlights the contrast between patients’ passive acceptance of medication prescriptions and the urge to actively experiment and adjust doses to optimize medication use in daily life. Finally, Taking oral medication for Type 2 diabetes: a unique context describes features specific to the Type 2 diabetes medication experience, including lack of symptoms and the perceived relationship between medication and diet, which may influence adherence.	Diabetes type 2 patients taking oral medication	8 studies
Nielsen et al., 2018	Adherence to medication in patients with chronic kidney disease: a systematic review of qualitative research	Denmark	To synthesize findings from qualitative studies of patients’ experiences of factors that facilitate and hinder adherence to medication.	Systematic review	MEDLINE, Embase, and CINAHL	Three analytical themes with the subthemes; (1) logistics (establishing and maintaining routines, and the costs of buying medication), (2) benchmarking the need for medication (absence of effect from a lay perspective, lacking understanding of medication indications and effects and being spurred by emergent symptoms) and (3) the quality of the patient- physician relationship (eliciting patients’ wishes for involvement in decisions concerning medication and lacking information).	Adult patients with chronic kidney disease	381 (19 studies)
Rashid et al., 2014	Medication taking in coronary artery disease: A systematic review and qualitative synthesis	UK	To understand from a patient perspective the factors that promote medication persistence.	Systematic review	MEDLINE, Embase, Psy- cINFO, SCOPUS, CINAHL, ASSIA, and SSCI	Some patients hold fatalistic beliefs about their disease, whereas others believe they have been cured by interventions; both can lead to failure to take medication. Patients who adapt to being a “heart patient” are positive about medication taking. Some individuals dislike taking tablets generally and are wary of long-term effects. Relationships with prescribing clinicians are of critical importance for patients, with inaccessibility and insensitive terminology negatively affecting patients’ perceptions about treatments.	Adult patients with cardiovascular disease	391 (17 studies)
Rathbone et al., 2017	A systematic review and thematic synthesis of patients’ experience of medicines adherence	UK	To explore patients’ lived experiences of medicines adherence reported in the phenomenological literature, through systematic review and thematic synthesis	Systematic review	CINAHL, PsychInfo, Web of Science, Sociological Abstracts, MEDLINE	Descriptive themes identified included (1) dislike for medicines, (2) survival, (3) perceived need, including (a) symptoms and side-effects and (b) cost, and (4) routine. Analytic themes identified were (1) identity and (2) interaction.	Adult patients with cardiovascular disease	463 (22 studies)
Tong et al., 2011	The perspectives of kidney transplant recipients on medicine taking: A systematic review of qualitative studies	Australia	To summarise and synthesise published qualitative studies on the experiences, perspectives, beliefs and attitudes of kidney transplant recipients on medicine taking.	Systematic review	Medline, PsycINFO, EMBASE, Cochrane Database from inception until Week 3 of January 2010	(1) attitudes towards medicine taking, its impact on lifestyle, self-image, relationships and outlook on life; (2) inadvertent forgetfulness, preoccupation with life commitments; (3) medication properties; (4) structure of healthcare services, poor access to pharmacy or affordable medications and conflicting medical appointments; (5) personal efforts in managing medications, organizing and devising strategies for taking medicines on time; and (6) availability of external social support	Adult patients with kidney transplants	207 (7 studies)
